# The Role of Emotional Intelligence in Engagement in Nurses

**DOI:** 10.3390/ijerph15091915

**Published:** 2018-09-03

**Authors:** María del Carmen Pérez-Fuentes, María del Mar Molero Jurado, José Jesús Gázquez Linares, Nieves Fátima Oropesa Ruiz

**Affiliations:** 1 Department of Psychology, Faculty of Psychology, University of Almería, Almería 04120, Spain; mmj130@ual.es (M.M.M.J.); sej473@ual.es (N.F.O.R.); 2 Department of Psychology, Universidad Autónoma de Chile, Providencia 7500000, Chile; jlinares@ual.es

**Keywords:** engagement, emotional intelligence, nurses

## Abstract

Aware that engagement in the healthcare field needs high levels of emotional intelligence, we began this study to determine relationship between engagement and emotional intelligence in nurses. The objective of this study was to determine the explanatory value of the components of emotional intelligence for engagement in a sample of nurses. The final study sample was made up of 2126 working nursing professionals. Data was obtained by distributing, an ad hoc questionnaire was used to collect sociodemographic information, and to collect professional and employment information, the Utrecht Work Engagement Scale and the Reduced Emotional Intelligence Inventory for Adults were used. The results showed that nurses with higher levels of emotional intelligence also scored more highly in engagement, with the interpersonal factor being the greatest predictor of engagement. This study has significant practical implications for the creation of intervention programs and activities to improve the performance of nurses in the workplace.

## 1. Introduction

Professional practice in healthcare requires a lot of personal and organizational engagement. Nurses perform many different care and treatment activities with the primary aim of contributing to the promotion, stabilization, and maintenance of their patients’ health. Using a broad concept of health [1], understood as a state of complete physical, mental, and social well-being and not merely the absence of disease or infirmity, engagement becomes a fundamental variable for quality patient care [2,3,4].

Engagement has been empirically shown to influence nursing performance, with a consequent impact on health care results [5]. From a psychological point of view, engagement leads to subjective wellbeing [6] as it allows an individual to enter a flow state [7] and satisfy basic psychological needs of autonomy and competence [8,9]. Previous research in nursing has confirmed a positive relationship between engagement and self-efficacy as well as job satisfaction [10,11]. Research has also found significant associations between engagement and personal factors such as mental health, locus of control, and job satisfaction [12,13].

Engagement has been defined by three fundamental dimensions: vigor, dedication, and absorption [14]. Vigor is characterized by high levels of energy and mental resilience in the face of difficulties and implies effort and persistence at work. Dedication is defined as being closely involved in one’s work, has a cognitive dimension or belief in what one is doing, and an affective dimension, related to feelings of enthusiasm, inspiration, pride, challenge, and significance. Absorption is characterized by a state of abstraction at work, experiencing a feeling of enjoyment associated with the desire to keep working. In that respect, Maslach, Schaufeli, and Leiter [15] demonstrated a strong negative association between vigor and burnout, and between dedication and indifference to work performance, indicating these dimensions as respective polar opposites. However, absorption is not the opposite to a lack of professional efficacy, and are two distinct concepts [16].

Studies comparing engagement by gender have produced controversial results, from those confirming the existence of significant differences [17,18,19,20,21] to those which found no differences [22] or differences with a small effect size [18]. Where differences have been found in engagement according to sex, the results have not been conclusive. On the one hand, Schaufeli and Bakker [20] found that men exhibited greater general engagement and higher levels of dedication and absorption than women, whereas in another study [19] the opposite was found, with women scoring higher than men in overall engagement, and in absorption and dedication. Various researchers have found that women scored significantly higher than men in vigor [17,19,23]. In samples of nurses, age has been found to be positively related to engagement, although the associations were weak [18].

We may deduce from this that engagement is part of a nurse’s value system, and should be an important objective from an organizational point of view. Personal effort and identification with the task can lead healthcare professionals to experience positive emotions and exhibit greater satisfaction in patient care. For that reason, if engagement is a fundamental pillar of patient care, positive emotions and emotional intelligence (EI) must also be fundamental.

Bar-On [24] stated that emotional intelligence referred to a variety of non-cognitive skills, competencies, and abilities that influenced a person’s capacity to succeed in the face of daily demands and pressures. Being emotionally intelligent implies the ability to address, understand, and feel one’s own emotions and those of others, and being able to respond and act accordingly (intrapersonal, interpersonal, stress management, adaptability, and general mood). In a healthcare context, emotional intelligence has been related to lower levels of stress and job satisfaction [25,26,27,28].

In terms of sex-related differences, Liébana et al. [23] found that women scored significantly higher than men in emotional intelligence. In research analyzing each component of emotional intelligence separately [29], female nurses scored higher than male nurses in the interpersonal dimension. However, others [30] found no significant relationship between sex and scores in the interpersonal dimension in a sample of dental students.

Research into the relationship between other dimensions of emotional intelligence and gender has produced contradictory results. Van Dusseldorp et al. [31] found that female nurses scored significantly higher than male nurses in some aspects related to intrapersonal factors. However, in another study [29], male nurses scored higher than female nurses in intrapersonal components and stress management. Similarly, others [30] found higher scores in male nurses’ intrapersonal components, stress management, and mood when compared to female nurses. In addition, in other cases [32] it was found that men scored significantly higher in the adaptability dimension.

Age was not found to be associated with the emotional intelligence of nurses [31], which was in contrast to other results [33], who found that nurses’ empathy diminished with age. One study [34] found no significant differences between the emotional intelligence scores in nurses based on demographic variables such as age, sex, marital status, or having children.

Research on the relationship between engagement and emotional intelligence in teachers [35], in healthcare [36], etc. One study [36] found that nurses with higher levels of emotional intelligence or better opinions of organizational fairness tended to exhibit greater levels of engagement. In this same study, the four emotional intelligence dimensions were found to be positively correlated with engagement. In another study, [37] found that personal resources such as emotional competence were closely related to engagement in nursing, whereas a study of nurses’ perceptions about the skills they need to do their jobs successfully showed social intelligence to be a predictor of engagement [11]. Some authors [38] suggested that people who were not emotionally intelligent would not be able to deal with the demands of their jobs and would be more likely to succumb to burnout and reduced commitment, which would end up affecting their wellbeing at work.

Starting from these premises, and aware that engagement in the healthcare field needs high levels of emotional intelligence, we began this study into the relationship between engagement and emotional intelligence in nurses. We proposed the following objective: to determine the explanatory value of the components of emotional intelligence for engagement in a sample of nurses.

We began with the following hypotheses: (1) despite the literature review not producing conclusive evidence, we expected to find differences in emotional intelligence and engagement according to sociodemographic variables, principally sex and age; (2) we expected to find significant positive correlations between emotional intelligence and engagement in nurses; and (3) the emotional intelligence dimensions of stress management, mood, and interpersonal factor will have the greatest predictive value for engagement in nurses.

## 2. Materials and Methods

### 2.1. Participants

The initial sample was made up of 2218 nurses from Andalucía (Spain) who were randomly selected from various centers. We identified 92 cases that were removed from the sample for not completing the whole questionnaire (32 subjects) or because we found that they had completed it randomly (60 subjects). As the main variable in the study was engagement, the selection of participants included noting their current working situation (permanent or temporary contracts). The resulting sample was made up of 2126 working nursing professionals (69.6% with temporary contracts, *n* = 1479, and 30.4% with permanent contracts, *n* = 647).

The mean age of the participants was 31.66 years old (*SD* = 6.66), ranging between 22 and 60 years old. Over three-fifths (84.9%, *n* = 1479) were women and 15.1% (*n* = 321) were men. Just over two-thirds of the participants (69.7%, *n* = 1482) had no children, 13.3% (*n* = 284) had one child, 14.4% (*n* = 306) had two children, and 2.5% (*n* = 54) had three or more children.

### 2.2. Instruments

We created an ad hoc questionnaire to collect sociodemographic data (age, sex, number of children, type of work contract).

The Utrecht Work Engagement Scale (UWES) [20]; is a self-reporting scale to evaluate engagement at work through 17 items with a seven-point Likert-type response scale. It produces information about three aspects of engagement: vigor (e.g., “At my work, I feel bursting with energy”), dedication (e.g., “I find the work that I do full of meaning and purpose”), and absorption (e.g., “Time flies when I’m working”). The scale gives an overall engagement score and a score for each of the specific dimensions. This instrument has demonstrated appropriate reliability and validity [14]. In our sample of nurses, the internal reliability indices in each of the dimensions were very good, with a Chronbach’s alpha of 0.84 in the vigor dimension, 0.89 in dedication, and 0.81 in absorption.

The Reduced Emotional Intelligence Inventory for Adults (EQ-i-20M) [39] was validated and assessed by the authors for the adult Spanish population, and derived from the adaptation for adults of the Emotional Intelligence Inventory: Young Version (EQ-i-YV) from Bar-On and Parker [40]. It consists of 20 items with four response alternatives in Likert-type scales. It was structured as five factors: intrapersonal (area that includes the following components: emotional understanding, of self, assertiveness, self-concept, self-realization, and independence; e.g., “I can describe my feelings easily”); interpersonal (empathy, social responsibility and interpersonal relationship; e.g., “I understand well how other people feel”); stress management (stress tolerance and impulse control; e.g., “I find it hard to control my anger”); adaptability (proof of reality, flexibility and problem solving; e.g., “I can solve problems in different ways”); and general mood (happiness and optimism; e.g., “I feel good about myself”). Cronbach’s alpha for each of the scales was: 0.90 for intrapersonal; 0.75 for interpersonal; 0.82 for stress management; 0.82 in adaptability; and 0.87 for general mood.

### 2.3. Procedure

Prior to collecting data, we assured the participants that the treatment of data in the study would comply with applicable standards of data security, confidentiality, and ethics. The study was approved by the Bioethics Committee of the University of Almería (Spain) (ethic code: UALBIO2017/011). The application of the questionnaire was done through a web platform which allowed subjects to complete them online. A series of control questions were included to monitor for random or incongruent responses, which were removed from the study.

### 2.4. Data Analysis

We confirmed the univariate normality of the sample following the criteria in which the maximum allowed values for asymmetry and kurtosis are 2 and 7 respectively, and the multivariate normality whit the use of Kolmogorov–Smirnov, obtaining values greater than *p* < 0.05 in all the variables. We first analyzed sociodemographic variables such as gender, age, and number of children. To identify significant differences between men and women, we used the Student’s *t*-test for independent samples of the components of emotional intelligence and for each dimension of engagement. In order to identify the relationships between those variables and the subjects’ ages and numbers of children, we calculated the Pearson correlation coefficient.

In order to understand how the predictor variables (emotional intelligence: intrapersonal, interpersonal, stress management, adaptability, and general mood) related to the criterion variable (engagement: vigor, dedication, and absorption), we performed a stepwise multiple linear regression analysis. Finally, we performed a nonlinear predictive CHAID (chi-square automatic interaction detector) regression and constructed a classification tree. In order to do so, we used the median engagement score (Md = 11.67) from all items. Scores below 11.67 were included in the low engagement group, and scores greater than or equal to 11.67 were included in the high engagement group. All analyses were performed using SPSS ver. 23.0 (IBM Corp, Armonk, NY, USA) statistical software for Windows. Finally, to identify mediation models for estimating the effects on engagement dimensions, a simple moderation analysis is carried out for each of the cases. To do this, the SPSS macro was used to compute models of simple moderation effects [41]. In addition, the bootstrapping technique with estimated coefficients from 5000 bootstrap samples was applied.

## 3. Results

### 3.1. Emotional Intelligence, Engagement, and Sociodemographic Variables

Table 1 shows the descriptive statistics for the sample as a whole and according to sex. It shows statistically significant differences, in some of the emotional intelligence components: intrapersonal: *t*_(2124)_ = −4.315, *p* < 0.001; interpersonal: *t*_(2124)_ = −4.609; *p* < 0.001; and adaptability: *t*_(2124)_ = 2.040; *p* < 0.05.

There were significant differences between the sexes in the engagement dimensions: vigor (*t*_(2124)_ = −3.131; *p* < 0.01), dedication (*t*_(2124)_ = −2.843; *p* < 0.01), and absorption (*t*_(2124)_ = −3.532; *p* < 0.001) with women who scored higher than men, in all cases.

Age was negatively correlated with the emotional intelligence interpersonal factor (*r* = −0.05; *p* < 0.01) and positively correlated with stress management (*r* = 0.05; *p* < 0.01). The three engagement dimensions were negatively correlated with age (vigor: *r* = −0.04, *p* < 0.05; dedication: *r* = −0.05, *p* < 0.01; absorption: *r* = −0.05; *p* < 0.01).

Finally, we found negative correlations between the number of children with the emotional intelligence Interpersonal factor (*r* = −0.06, *p* < 0.01), and with the engagement dimensions of vigor (*r* = −0.04, *p* < 0.05) and (*r* = −0.05, *p* < 0.05) absorption.

### 3.2. Components of Emotional Intelligence as Predictors of Engagement in Nurses

The correlation coefficients we calculated showed that nurses with high levels of emotional intelligence also exhibited higher scores in engagement. The correlation analysis showed that all of the emotional intelligence components were positively correlated with each of the engagement dimensions, with correlation indices ranging from *r* = 0.15 to *r* = 0.40, and *p* < 0.001 in all cases.

Table 2 shows that the regression analysis for the engagement dimension vigor gave four models, the fourth of which demonstrated the greatest explanatory power, with 22.8% (*R*^2^ = 0.228) of the variance explained by the factors in the model. To confirm the validity of the model, we analyzed the independence of the residuals. The Durbin–Watson *D* statistic was *D* = 1.964, confirming the absence of positive or negative autocorrelation. The value of *t* was associated with a probability of error of less than 0.05 in all of the included variables in the model. The standardized coefficients showed that the variable with the greatest explanatory weight was the interpersonal factor. Lastly, the values of tolerance indicators and VIF suggested the absence of collinearity between the variables included in the model.

The analysis of the dedication component produced four models, the fourth of which demonstrated the greatest explanatory power, with 21.9% (*R*^2^ = 0.219) of the variance explained. The Durbin–Watson statistic confirmed the validity of the model (*D* = 1.941). The value of *t* was associated with a probability of error of less than 0.05 in all of the included variables in the model. The standardized coefficients indicated that general mood was the strongest predictor of dedication in the sample. The values of tolerance indicators and VIF suggested the absence of collinearity between the variables included in the model.

For the absorption dimension, the regression analysis produced four models, the fourth of which accounted for 14% of the explained variance (*R*^2^ = 0.140) with *D* = 1.961, confirming the validity of the model. The value of *t* was associated with a probability of error of less than 0.05 in all of the included variables in the model. In this case, the interpersonal component of emotional intelligence was the strongest predictor of absorption. The values of tolerance indicators and VIF suggested the absence of collinearity between the variables included in the model.

**Figure 1:**
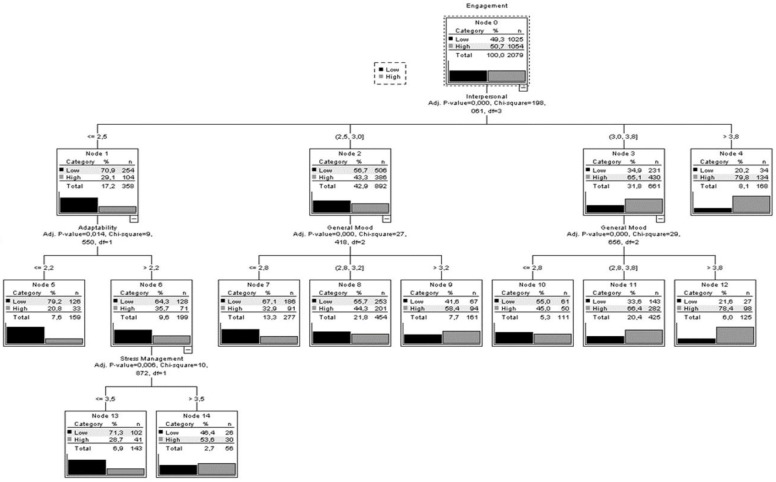
Regression and classification tree for engagement.

The decision tree (Figure 1) showed that the interpersonal factor was the best predictor of engagement. Participants with low scores in the interpersonal factor and low adaptability exhibited low levels of engagement (79.2%). High levels of engagement were present in those with high scores in the interpersonal variable (79.8%). Finally, the goodness of fit of the model functioning could be seen in its correct classification of 65.7% of the participants.

### 3.3. Mediation Models for Estimating the Effects on Engagement Dimensions

Based on the results of the regression analysis, the emotional intelligence interpersonal factor was taken as the independent predictor variable and mood as the mediating variable. Thus, three simple mediation models were computed with the interpersonal factor as the independent variable in all cases: in the first model, the dependent variable was ‘vigor’, in the second ‘dedication’, and in the third, ‘absorption’ was taken as the dependent variable.

**Figure 2:**
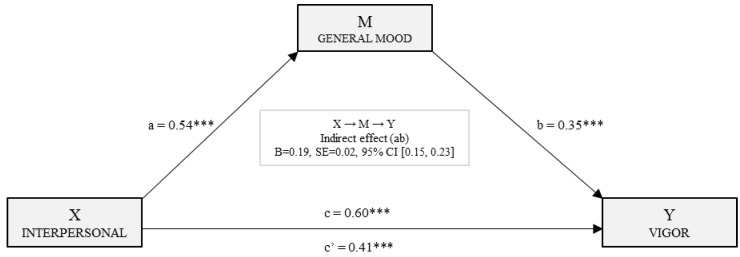
Simple mediation model of mood on the relationship between the interpersonal factor and the vigor dimension of engagement. (Note: ***p < 0.001).

Figure 2 shows the simple mediation model for vigor, including the direct, indirect, and total effects. In the first place, it may be observed that there was a statistically significant effect (*B*_Inter_ = 0.54, *p* < 0.001) of the interpersonal factor (X) on mood (M). The second regression analysis includes the interpersonal factor (X) and mood (M) in the equation. In both cases statistically significant effects on the dependent variable were found (vigor): M→Y (*B*_E_ánimo_ = 0.35, *p* < 0.001) and X→Y (*B*_Inter_ = 0.41, *p* < 0.001). With the third regression analysis, the total effect of the independent variable (X) on the dependent variable (Y) was estimated. In this case, a statistically significant effect of the interpersonal factor on the vigor dimension of engagement was found (*B*_Inter_ = 0.60, *p* < 0.001). Finally, the analysis of the indirect effect was carried out using bootstrapping, finding data supporting a significant level (*B* = 0.19, SE = 0.02, 95% CI (0.15, 0.23)).

**Figure 3:**
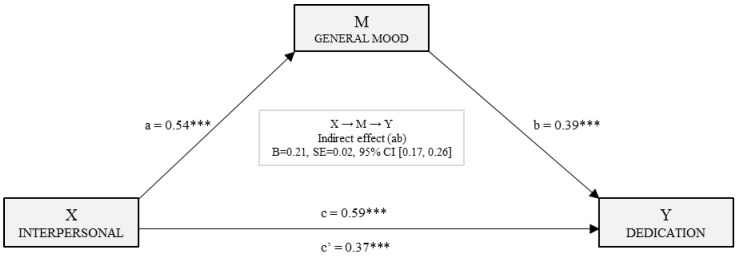
Simple mediation model of mood on the relationship between the interpersonal factor and the dedication dimension of engagement. (Note: ***p < 0.001).

Figure 3 shows the simple mediation model for dedication. Based on the second regression analysis, the effects of the independent variable (interpersonal) and the mediator (mood) on the dependent variable (dedication) were estimated. It may be observed that in both cases, the effect on dedication was statistically significant: (*B*_Inter_ = 0.37, *p* < 0.001) and (*B*_E_ánimo_ = 0.39, *p* < 0.001). The total effect of the Interpersonal factor on dedication was significant (*B*_Inter_ = 0.59, *p* < 0.001). Finally, with the analysis of the indirect effect with bootstrapping, data extracted supported a significant level (*B* = 0.21, SE = 0.02, 95% CI (0.17, 0.26)).

**Figure 4:**
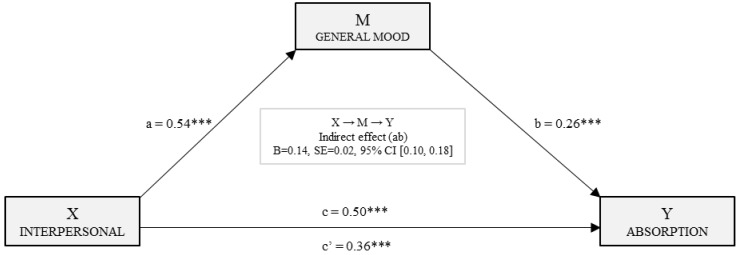
Simple mediation model of mood in the relationship between the interpersonal factor and the absorption dimension of engagement. (Note: ***p < 0.001).

Figure 4 shows the simple mediation model for absorption. With the second regression analysis, the effect of the independent variable was estimated taking absorption (Y) as the resulting variable, the effect of the independent variable (*B*_Inter_ = 0.36, *p* < 0.001) and the mediator (*B*_E_ánimo_ = 0.26, *p* < 0.001) were estimated, resulting statistically significant in both cases. The total effect of the interpersonal factor on absorption was significant (*B*_Inter_ = 0.50, *p* < 0.001). Finally, with the analysis of the indirect effects with bootstrapping, a significant effect was found (*B* = 0.14, SE = 0.02, 95% CI (0.10, 0.18)).

## 4. Discussion

This study achieved our initial objective by determining the explanatory value of the components of emotional intelligence in engagement in a sample of nurses.

We found that women exhibited higher levels of emotional intelligence in some emotional intelligence components (interpersonal and intrapersonal). These findings agreed with our second hypothesis, where we expected to find significant, positive correlations between emotional intelligence and engagement in nurses. Other research has produced similar results, especially regarding the relationship between the interpersonal [29,31,32] and the intrapersonal dimensions of emotional intelligence [30,31].

Some studies have found higher scores in men for the dimensions of stress management [29], general mood [30], and adaptability [32]. However, it is important to note that men were over-represented in the samples in those studies [29,32], and in order to draw more safely definitive conclusions, it would be necessary to have samples that were more evenly balanced between men and women.

Our results showed that age was negatively correlated with the interpersonal factor of emotional intelligence and positively correlated with stress management. Harper and Jones-Schenk [33] found that nurses’ empathy diminished with age. Kahraman and Hiçdurmaz [34] found a negative correlation between the number of children and the interpersonal factor of emotional intelligence, which was not in agreement with our results.

We found significant differences between the gender in all dimensions of engagement, with women scoring higher; this is in line with other research [19]. The three engagement dimensions were negatively correlated with age, which were similar to findings from other studies [16,18,20]. We also saw negative correlations between the numbers of children and the engagement dimensions of vigor and absorption.

We also achieved our objective of developing an explanatory model of engagement showing that nurses with higher levels of emotional intelligence also scored more highly in engagement, with the interpersonal factor being the greatest predictor of engagement. Other studies support the relationship between the two variables [11,35,36,37].

Thus, three simple mediation models were computed with the interpersonal factor as the independent variable in all cases: in the first model, the dependent variable was vigor; in the second dedication; and in the third, absorption was taken as the dependent variable. The analysis of the indirect effect was carried out using bootstrapping, finding data supporting a significant level the general mood in all cases.

Our results have significant practical implications for the creation of intervention programs and activities to improve the performance of nurses in the workplace (e.g., skills training programs for managing emotions in relationships with co-workers, patients). The results should, however, be considered with some care due to the following limitations. First, the data were gathered through online questionnaires completed by the nurses and may be biased as the subjects’ responses may be subject to desirability bias. Second, as the sample we used was very specific and limited to one type of profession in the healthcare field, it is possible that the results cannot be generalized to other related healthcare professions. Third, the study design did not allow us to determine whether the engagement and emotional intelligence scores remained constant over time. Finally, in Spain, nursing is a predominantly a female profession, which was reflected in the sample, and may be a limitation on the results.

Finally, we are continuing to work on the analysis of elements that encourage worker engagement, and future research should address other variables related to the subject (personality, self-esteem, etc.) and the work environment (such as number of patients dealt with, shift patterns, etc.) in order to continue describing this construct.

## 5. Conclusions

The results showed that there were significant differences in emotional intelligence and engagement when we looked at the sociodemographic variables in the study (sex, age, number of children). These findings supported our first research hypothesis, where we expected to find differences in emotional intelligence and engagement according to sociodemographic variables, although the results found in the reviewed literature varied.

Emotional intelligence explained 22.8% of the variability in the engagement dimension vigor, with the interpersonal factor having the greatest explanatory weight. It explained 21.9% of the variability in the dedication dimension, with general mood being the strongest predictor, and explained 14% of the variability in the absorption dimension, with the interpersonal component being the strongest predictor.

## Figures and Tables

**Table 1:** Emotional intelligence and engagement. Descriptive statistics and *t*-test by sex.

		Total *n* = 2126		Men *n* = 321		Women *n* = 1805		*t*	Sig.
		*M*	*SD*	*M*	*SD*	*M*	*SD*		
Emotional Intelligence	Intrapersonal	2.62	0.698	2.46	0.690	2.65	0.696	−4.315 ***	0.000
	Interpersonal	3.06	0.501	2.94	0.530	3.08	0.493	−4.609 ***	0.000
	Stress Management	3.25	0.567	3.26	0.569	3.24	0.567	0.380	0.704
	Adaptability	2.91	0.526	2.96	0.527	2.90	0.526	2.040 *	0.042
	General Mood	3.08	0.599	3.11	0.607	3.08	0.598	0.871	0.384
Engagement	Vigor	3.85	0.771	3.72	0.808	3.87	0.762	−3.131 **	0.002
	Dedication	4.07	0.794	3.94	0.884	4.09	0.775	−2.843 **	0.005
	Absorption	3.52	0.800	3.38	0.849	3.55	0.788	−3.532 ***	0.000

* The correlation is significant at 0.05; ** The correlation is significant at 0.01; *** The correlation is significant at 0.001.

**Table 2:** Multiple stepwise linear regression model (*N* = 2126).

Vigor	**Model**	***R***	***R*^2^**	**Adjusted *R*^2^**	**Change Statistics**					**Durbin Watson**
					**Standard Error of Estimation**	**Change in *R*^2^**	**Change in *F***	**Sig. of Change in *F***		
	1	0.397	0.158	0.158	0.708	0.158	398.401	0.000		1.964
	2	0.465	0.216	0.216	0.683	0.058	158.160	0.000		
	3	0.473	0.224	0.223	0.680	0.008	20.508	0.000		
	4	0.477	0.228	0.226	0.678	0.004	10.834	0.001		
	**Model 4**		**Non-standardized Coefficients**		**Standardized Coefficients**		***t***	**Sig.**	**Collinearity**	
			***B***	**std. error**	**Beta**				**Tol.**	**VIF**
	(Constant)		1.134	0.118			9.593	0.000		
	General Mood		0.264	0.033	0.205		8.104	0.000	0.570	1.756
	Interpersonal		0.364	0.037	0.236		9.953	0.000	0.647	1.547
	Stress Management		0.128	0.028	0.094		4.629	0.000	0.879	1.138
	Adaptability		0.128	0.039	0.087		3.292	0.001	0.519	1.928
Dedication	**Model**	***R***	***R*^2^**	**Adjusted *R*^2^**	**Change Statistics**					**Durbin Watson**
					**Standard Error of Estimation**	**Change in *R*^2^**	**Change in *F***	**Sig. of Change in *F***		
	1	0.407	0.165	0.165	0.726	0.165	420.764	0.000		1.941
	2	0.458	0.210	0.209	0.706	0.044	119.178	0.000		
	3	0.466	0.217	0.216	0.703	0.007	19.051	0.000		
	4	0.467	0.219	0.217	0.703	0.002	4.800	0.029		
	**Model 4**		**Non-Standardized Coefficients**		**Standardized Coefficients**		***t***	**Sig.**	**Collinearity**	
			***B***	**std. error**	**Beta**				**Tol.**	**VIF**
	(Constant)		1.391	0.122			11.449	0.000		
	General Mood		0.336	0.031	0.253		10.688	0.000	0.655	1.526
	Interpersonal		0.354	0.035	0.223		10.026	0.000	0.745	1.343
	Stress Management		0.130	0.029	0.093		4.510	0.000	0.875	1.142
	Intrapersonal		0.054	0.025	0.048		2.191	0.029	0.774	1.292
Absorption	**Model**	***R***	***R*^2^**	**Adjusted *R*^2^**	**Change statistics**					**Durbin Watson**
					**Standard Error of Estimation**	**Change in *R*^2^**	**Change in *F***	**Sig. of Change in *F***		
	1	0.316	0.100	0.100	0.759	0.100	236.011	0.000		1.961
	2	0.362	0.131	0.130	0.746	0.031	76.359	0.000		
	3	0.369	0.136	0.135	0.744	0.005	12.570	0.000		
	4	0.374	0.140	0.138	0.743	0.003	8.322	0.004		
	**Model 4**		**Non-Standardized Coefficients**		**Standardized Coefficients**		***t***	**Sig.**	**Collinearity**	
			***B***	**std. error**	**Beta**				**Tol.**	**VIF**
	(Constant)		1.361	0.128			10.598	0.000		
	Interpersonal		0.325	0.037	0.204		8.725	0.000	0.745	1.343
	General Mood		0.202	0.033	0.152		6.093	0.000	0.655	1.526
	Intrapersonal		0.098	0.026	0.086		3.743	0.000	0.774	1.292
	Stress Management		0.088	0.030	0.062		2.885	0.004	0.875	1.142
